# Genetic analysis of fetal skeletal dysplasia via whole exome sequencing and non-invasive prenatal diagnosis

**DOI:** 10.1080/07853890.2025.2606517

**Published:** 2025-12-28

**Authors:** Lulu Wang, Yinglu Zhang, Fangxiu Zheng, He Dong, Xinmei Zhu, Chenbo Jia, Bin Zhang

**Affiliations:** Department of Medical Genetics, Changzhou Maternal and Child Heath Care Hospital, Changzhou Medical Center, Nanjing Medical University, Changzhou, Jiangsu, China

**Keywords:** Skeletal dysplasia, genetic testing, whole exome sequencing, non-invasive prenatal diagnosis

## Abstract

**Objective:**

Fetal skeletal dysplasia (SD) is a complex group of bone and cartilage with high genetic heterogeneity and phenotypic diversity. Our study aimed to identify the genetic causes of fetal SD, provide an accurate prenatal diagnosis for those families investigate non-invasive prenatal detection strategy, and facilitate clinical diagnosis of fetal SD.

**Materials and Methods:**

A Total 34 fetuses with SD were recruited and analyzed using chromosomal microarray analysis (CMA) and whole exome sequencing (WES). Moreover, a non-invasive prenatal diagnosis (NIPD) based on next-generation sequencing (NGS) using circulating fetal DNA in maternal plasma was performed on 5 SD fetuses.

**Results:**

A Total 55.88% (19/34) fetueses with SD were identified with the genetic etiologies. A Total 27 cases underwent karyotype and CMA analysis, and the diagnostic rate was 3.70% (1/27), while 34 cases accepted WES, and the diagnostic rate was 55.88% (19/34). Of the 19 positive cases, 2 cases were identified with pathogenic CNVs (10.53%, 2/19), and the other 17 cases were identified with pathogenic or likely pathogenic variations (89.47%, 17/19). The FGFR3 was the most prevalent SD-causing gene (47.06%, 8/17). In addition, 5 cases accepted the NIPD based on NGS for the detection of fetal SD, and the results were all consistent with those of amniocentesis.

**Conclusions:**

Our study highlights the advantages of WES compared with CMA in genetic diagnosis in fetal SD. Furthermore, our study revealed the excellent detection efficiency of NIPD based on NGS, which may be a potential noninvasive detection method in fetal SD.

## KEY MESSAGES

- We identified the genetic causes of fetal SD and provided a accurate prenatal diagnosis for those families.
- Our results highlighted the advantages of WES compared with CMA in genetic diagnosis in fetal SD.
- Our study revealed the excellent detection efficiency of NIPD based on NGS, which may be a potential noninvasive detection methods in fetal SD.

## 1. Introduction

Fetal skeletal dysplasia (SD) is a complex group of bone and cartilage disorders characterized by abnormal shape, size, density, and integrity of the skeleton. The incidence of SD is estimated to be approximately 2.4–4.5 per 10,000 births [1]. The clinical manifestations vary form mild to severe abnormalities with perinatal mortality due to respiratory distress and lung failure [2]. SD are often caused by genetic and/or environmental factors, and monogenic gene mutations are the main factors of fetal SD. According to the 2023 Nosology and Classification of Genetic Skeletal Disorders, 771 types of bone diseases classified into 41 groups, including 552 genes, have been described and defined by molecular, biochemical, and radiographic criteria [3].

Traditionally, the prenatal diagnosis of fetal SD mostly relies on ultrasound (US), and the sensitivity is reported to be approximately 40 ∼ 60% [4]. The US has severe limitations in differentiating numerous types of SD owing to the overlapping features and phenotypic variability. The lack of an accurate and precise prenatal diagnosis of fetal SD makes prenatal counselling and pregnancy management challenging, while molecular diagnosis offers the possibility to identify the specific cause of SD. The conventional molecular diagnosis methods include karyotyping and chromosomal microarray analysis (CMA), with a total diagnostic yield of 36% [5,6]. Despite the advances in molecular diagnostics, less than half of fetal SD can be diagnosed. Additionally, the majority of SD are caused by monogenic gene mutations, which can not be diagnosed through karyotyping and CMA. It is still challenging to establish a specific diagnosis strategy due to the high genetic heterogeneity and phenotypic diversity of SD. With the advantage of sequencing all exons containing over 85% of the genetic variants associated with human genetic diseases, the whole exome sequencing (WES) has been a helpful tool to identify and differentiate the genetic cause of SD [7]. However, the reports about prenatal diagnosis of fetal SD using WES are still limited, and the sample size of most studies were relatively small.

For genetic testing, fetal specimens are typically obtained through invasive procedures such as chorionic villus sampling and amniocentesis, which are not easily accepted by pregnant women owing to the risk of miscarriage or infection. The discovery of fetal cell-free DNA (cf-DNA) circulating in maternal blood samlpes has opened a promising opportunity for safer, noninvasive detection of fetal genetic defects. Some molecular assays have been developed for non-invasive detection of fetal SD, such as thanatophoric dysplasia and achondroplasia [8,9]. Most of these assays are based on polymerase chain reaction (PCR) and restriction fragment length polymorphism analysis (RFLP), which require a large volume of maternal plasma and can only detect limited loci simultaneously [10]. The development of targeted capture sequencing technology provides the possible simultaneous analysis of many candidate genes. The application of non-invasive prenatal diagnosis (NIPD) based on next-generation sequencing (NGS) of achondroplasia and thanatophoric dysplasia has been reported [11]. However, the NIPD based on NGS for SD is still in its infancy, and the specificity and sensitivity of it are unclear.

In this study, a total 34 fetuses with SD were recruited and analyzed using CMA and WES. Moreover, an NIPD based on NGS using circulating fetal DNA in maternal plasma was performed on 5 SD fetuses. Our study aimed to identify the genetic causes of fetal SD, provide an accurate prenatal diagnosis for those families, and evaluate the diagnostic yield of prenatal WES and the NIPD based on NGS for fetal SD.

## 2. Materials and methods

### 2.1. Subjects

A total of 34 families with SD fetuses based on clinical and sonographic diagnosis were recruited at the Department of Medical Genetics, Changzhou Maternal and Child Health Care Hospital between January 2019 and September 2024. The study adhered to the Declaration of Helsinki and was approved by the Ethics Committee of the Changzhou Maternal and Child Health Care Hospital (No. 2018006). Written informed consents were obtained from all parents of the fetuses for the study.

### 2.2. Sample collection

We collected 26 amniocytes and 8 fetal abortion tissues. Amniotic fluid (30 ml) was collected by amniocentesis under ultrasound-guided procedures from 26 pregnant women who accepted the prenatal diagnosis. Fetal abortion tissues were taken from the outer thigh skin (2 × 2 cm) of 8 fetuses after the termination of pregnancy. Peripheral blood samples (5 ml) were obtained from parents and stored in EDTA blood tubes (Becton Dickinson and Company, United Kingdom). Genomic DNA was extracted from amniotic fluid using the QIAamp DNA Mini Kit (Qiagen, China) according to the manufacturer’s protocol. Maternal plasma was obtained from the peripheral blood samples of four pregnant women by a double centrifugation procedure at 1,600 × g for 10 min at 4 °C with 8 h after blood collection. The cf-DNA was extracted using the QIAamp Circulating Nucleic Acid Kit (Qiagen, China) from the maternal plasma.

### 2.3. Karyotyping and chromosomal microarray analysis (CMA)

Amniotic fluid (20 ml) was used for cell culture and karyotyping. Five metaphase cells were examined to detect the structural chromosomal abnormalities, and at least 15 metaphase chromosomes were examined to detect the numerical abnormalities of chromosomes using G-banding.

The CMA procedure was carried out as described previously [12]. After extracted, genomic DNA (250 ng) was then amplified, labeled, and hybridized to the GCS 3000Dx v.2 platform (Affymetrix, Santa Clara, CA, USA). The SNP array experiments were conducted using an Affymetrix CytoScan 750K microarray chip (Agilent, Santa Clara, California) which can detect CNVs at an effective minimal resolution of 100 kb and regions of allelic homozygosity (ROHs) at a threshold of 5 Mb. After hybridization with the fragmented DNA, the chip was washed with buffer and scanned with an Alaser scanner (Wilkes Optoelectronics, Shenzhen, China). The data was analyzed by Affymetrix Chromosome Analysis Suite v3.2 software (ChAS) (Affymetrix, Santa Clara, California). Pathogenicity of the potential copy number variations (CNVs) was evaluated according to the American College of Medical Genetics and Genomics (ACMG) guidelines and the Clinical Genome Resource (ClinGen) [13].

### 2.4. Whole exome sequencing (WES)

After extraction, DNA (300 ng) underwent shearing to produce 100–500 bp fragments, and 200–300 bp fragments were selective retained with VAHTS DNA clean beads (Vazyme, Nanjing, China). Subsequently, the sample library was hybrided with the customized gene fragment capture probes (KAPA HyperExome, Roche). The coding exons and flanking sequences of over 20,000 genes were captured and then sequenced on the MGISEQ-2000 platform (MGI, Shenzhen, China) according to the manufacturer’s protocols. The average sequencing depth of the target region was higher than 170×. The evaluation of the raw data, and the removal of low-quality and contaminated reads was performed using the SOAPnuke software. Then, the filtered clean reads were aligned to the human reference sequence (GRCh37/hg19) with the Burrows-Wheeler Aligner (BWA, version 0.7.12). Single nucleotide variants, deletions and insertions were called using the GATK software package (version 4.0). The screening and annotation of variants were carried out by the ANNOVAR software. The exon CNVs were detected using the ExomeDepth software, while the CNVs exceeding 1 M were detected using the CNV kit software. Variants were interpreted according to the ACMG and the ClinGen guidelines. The candidate variants identified by WES were confirmed by Sanger sequencing.

### 2.5. Non-invasive prenatal diagnosis (NIPD) by next-generation sequencing (NGS)

#### 2.5.1. Target region sequencing

cfDNA was sheared into 200–250 bp fragments and then underwent end-repair, phosphorylation, and A-tailing reactions. Adaptors with specific barcodes were ligated to the cfDNA fragments by PCR amplification. The construction of the cfDNA library was performed using the KAPA LTP Library Preparation Kit (Kapa Biosystems, Cape Town, United States). Next, hybridization capture was conducted by the NimbleGen. After quantitation, the post-capture library was sequenced (101 bp read) on the Hiseq 2500 system (Illumina, California, United States).

#### 2.5.2. Alignment and variant calling

SOAPnuke was used to trim and filter the raw data. Low-quality reads were removed using Picard tools (version 1.87, http://picard.sourceforge.net). The clean reads was aligned to the GRCh37/hg19 using BWA software. Variation calling was accomplished by Genome Analysis Toolkit (GATK) software (version 4.0). CoverageAnalysis (version 5.0.2.0.) was used to calculated the coverage, uniformity and on-target rate.

#### 2.5.3. Fetal fraction estimation and prediction of fetal genotype

The construction of parental haplotypes linked with pathogenic allele were established by the target region genotypes of trios. Fetal fraction (FF) in maternal plasma was calculated using SNPs that are homozygous in parents according to the following formula:

$$\text{FF}=2\times \frac{\sum d_{f}}{\sum {(d}_{f}+d_{m})}$$

where $d_{f}$ is the number of reads corresponding to the special allele of fetus, and $d_{m}$ is the number of reads corresponding to the allele shared by the mother and fetus.

Paternal or maternal inheritance was determined using the types of SNPs. Parental haplotype was constructed assisted hidden Markov model (HMM) and Viterbi algorithm using the sequencing data of maternal plasma to deduce the fetal genotypes of pathogenic sites. The results of NIPD was validated by Sanger sequencing.

## 3. Results

### 3.1. Clinical information and ultrasound findings

Among the 34 families in our study, the mean maternal age of the cohort was 28.7 years old, and the gestational week ranged from 17 to 26 weeks. All fetuses were divided into three groups according to the anomalies of US: (1) short limb malformations (fetal long bone length <-2SD of the reference ranges at mid-trimester ultrasonography, with or without abnormal shape of long bones, fracture, or thoracic hypoplasia (38.24%, 13/34)); (2) multiple malformations (abnormalities of the skeletal system with other additional anomalies of other systems (29.41%, 10/34)); (3) other SD which appeared without abnormalities of length, such as shapolydactyly, syndactyly, talipes equinovarus, and widened skull suture (32.35%, 11/34). Except for case 14, all cases were divided into the unilateral malformation group (12.12%, 4/33, cases 19, 25, 33, 34) and the bilateral malformation group (87.88%, 29/33). Detailed clinical information is shown in Table S1. Images of US examinations of some fetuses with SD are shown in Figures S1-S4.

**Figure 1.**
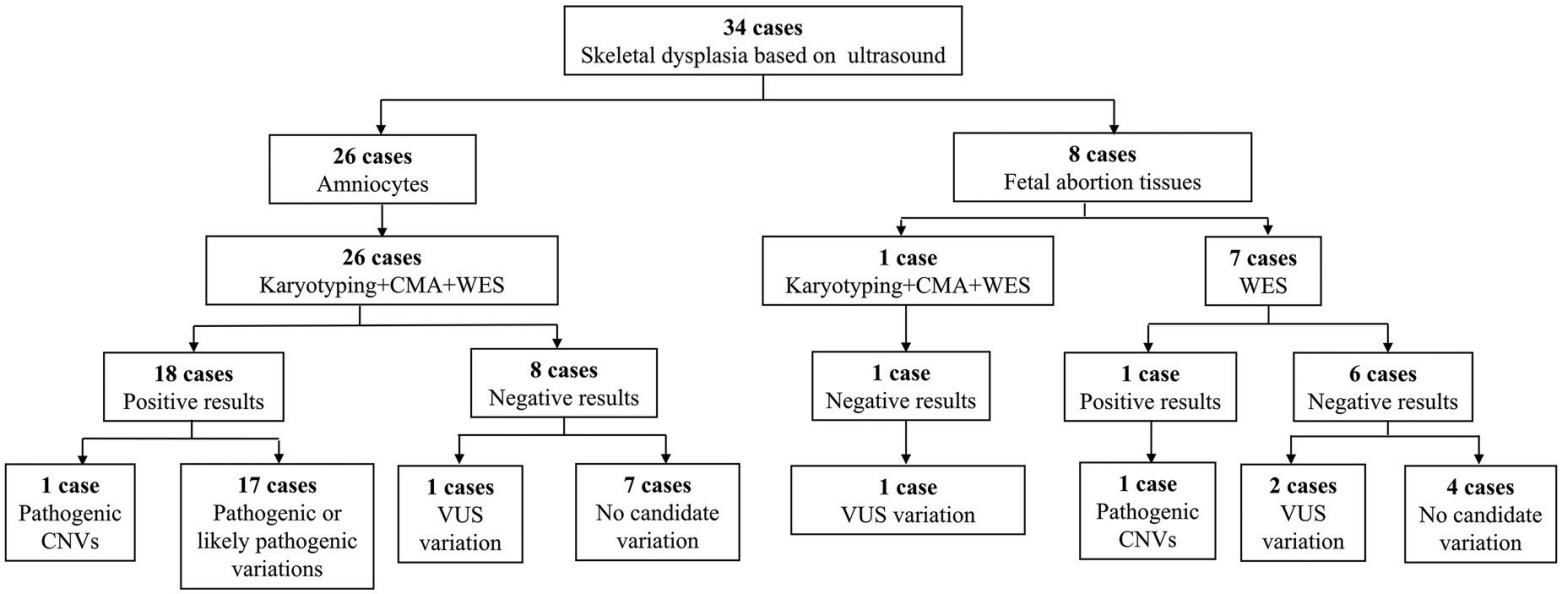
Flowchart of the methods and results of 34 cases in our study. CMA: chromosomal microarray analysis; WES: whole exome sequencing; CNVs: copy number variations; VUS: variation of uncertain significance.

### 3.2. Abnormalities by karyotype analysis and copy number variations (CNV)

A Total 27 cases underwent karyotype and CMA analysis. A Total 27 cases showed negative in the chromosome G band karyotype (Figure 1), and 26 cases showed negative in CMA except for Case 1, with a diagnostic rate of 3.70% (1/27). In case 1, we found a 4.9 Mb pathogenic heterozygous duplication in the chromosome 11 p15.5-p15.4.

Among three groups, the diagnostic rate of CMA in the multiple malformations group (10%, 1/10) was the highest, followed by short limb malformations group (7.69%, 1/13), and other SD group (0%, 0/11). The diagnostic rate of CMA in the unilateral malformation group was 0% (0/4), while that in the bilateral malformation group was 3.45% (1/29).

### 3.3. Abnormalities detected by whole exome sequencing (WES)

Ā Total 34 cases accepted WES, and 19 fetuses were confirmed with genetic etiologies, a diagnostic rate of 55.88% (19/34). Of the 19 positive cases, 2 cases were identified with pathogenic CNVs (10.53%, 2/19), and other 17 cases were identified with pathogenic or likely pathogenic variations (89.47%, 17/19).

Case 1 had a 4.9 Mb heterozygous duplication in the chromosome 11 p15.5-p15.4 (pathogenic, *de novo*). Case 30 had a 56.82 Mb heterozygous deletion in the chromosome X p22.33p11.21 (pathogenic, *de novo*) and a 98.10 Mb anchimerism (chimeric ratio 44%) in the chromosome X p11.21q28 (pathogenic, *de novo*). Of the remaining 17 positive cases, 11 cases showed autosomal dominant (AD) inheritance, while 6 cases showed autosomal recessive (AR) inheritance. Among 15 cases unconfirmed with genetic etiologies, 4 cases had a variation of uncertain significance (VUS) and 11 cases had no candidate variation (Table 1).

**Table 1.** Genetic analysis of 34 SD fetuses using WES.

Case	Gestational week	Malformation	CNV	Gene	NM	Variant	Protein change	Zygosity	Inheritance pattern	Variant origin	ACMG
1	23 + 3	Short limb malformations	dup (11) (p15.5-p15.4) 4.9 Mb					Het		NA	P
2	17 + 4	Short limb malformations		*FGFR3*	NM_000142.4	c.742C > T	p.Arg248Cys	Het	AD	*de novo*	P
3	25 + 2	Short limb malformations		*FGFR3*	NM_000142.4	c.742C > T	p.Arg248Cys	Het	AD	*de novo*	P
4	20 + 4	Short limb malformations		*FGFR3*	NM_000142.4	c.742C > T	p.Arg248Cys	Het	AD	*de novo*	P
5	15 + 4	Short limb malformations		*FGFR3*	NM_000142.4	c.742C > T	p.Arg248Cys	Het	AD	*de novo*	P
6	24 + 3	Short limb malformations		*FGFR3*	NM_000142.4	c.742C > T	p.Arg248Cys	Het	AD	*de novo*	P
7	17	Other skeletal dysplasia		*FGFR3*	NM_000142.4	c.742C > T	p.Arg248Cys	Het	AD	*de novo*	P
8	23 + 3	Short limb malformation		*FGFR3*	NM_000142.4	c.1138G > A	p.Gly380Arg	Het	AD	*de novo*	P
9	24 + 2	Multiple malformations		*FGFR3*	NM_000142.4	c.1948A > G	p.Lys650Glu	Het	AD	Mat	P
10	21 + 1	Multiple malformations		*COL1A1*	NM_000088.3	c.2110G > C	p.Gly704Arg	Het	AD	*de novo*	P
11	23 + 1	Other skeletal dysplasia		*COL1A2*	NM_000089.3	c.892G > A	p.Gly298Ser	Het	AD	Mat	P
12	24	Short limb malformations		*COL2A1*	NM_001844.4	c.2546G > T	p.Gly849Val	Het	AD	*de novo*	LP
13	25 + 4	Multiple malformations		*COL2A1*	NM_001844.4	c.3401G > C	p.Gly1134Ala	Het	AD	*de novo*	LP
14	24 + 2	Multiple malformations		*RUNX2*	NM_004348.4	c.581-4_581-1delGCAG	NA	Het	AD	Mat	P
15	24 + 4	Other skeletal dysplasia		*LMX1B*	NM_002316.3	c.139 + 1G > T	NA	Het	AD	Mat	LP
16	25 + 4	Other skeletal dysplasia		*GLI3*	NM_000168.5	c.1755_1756insTGC	p.Lys585_Ala586insCys	Het	AD	Pat	LP
17	22 + 5	Short limb malformations		*DYNC2H1*	NM_001080463.1	c.7594C > T	p.Arg2532Trp	Het	AR	Mat	LP
						c.10228-2A > G	NA	Het	AR	Pat	LP
18	24	Short limb malformations		*ALPL*	NM_000478.5	c.407G > A	p.Arg136His	Het	AR	Mat	LP
						c.1388C > A	p.Ser463Tyr	Het	AR	Pat	LP
19	22 + 4	Other skeletal dysplasia		*TBX4*	NM_018488.3	c.1518delC	p.Ser507Argfs*4	Het	AD/AR	Mat	VUS
20	23 + 3	Short limb malformations		–							
21	26	Other skeletal dysplasia		–							
22	25 + 1	Short limb malformations		–							
23	24	Other skeletal dysplasia		–							
24	23 + 6	Multiple malformations		–							
25	25 + 2	Other skeletal dysplasia		–							
26	24 + 1	Multiple malformations		–							
27	24 + 6	Short limb malformations		*FLNB*	NM_001457.4	c.4051G > A	p.Val1351Met	Het	AD/AR	NA	VUS
28	22 + 6	Multiple malformations		*COL2A1*	NM_001844.5	c.3447_3464del	p.Asp1150_Gly1155del	Het	AD	*de novo*	VUS
29	23 + 1	Other skeletal dysplasia		*TRPV4*	NM_021625.5	c.1795A > G	p.Thr599Ala	Het	AD	Pat	VUS
30	22	Multiple malformations	del(X)p22.33p11.21, 56.82 Mb; del(X)p11.21q28, 98.10 Mb					Het		*De novo*	P/P
31	24 + 4	Multiple malformations									
32	25	Multiple malformations									
33	23 + 4	Other skeletal dysplasia									
34	23 + 2	Other skeletal dysplasia									

**Figure 2.**
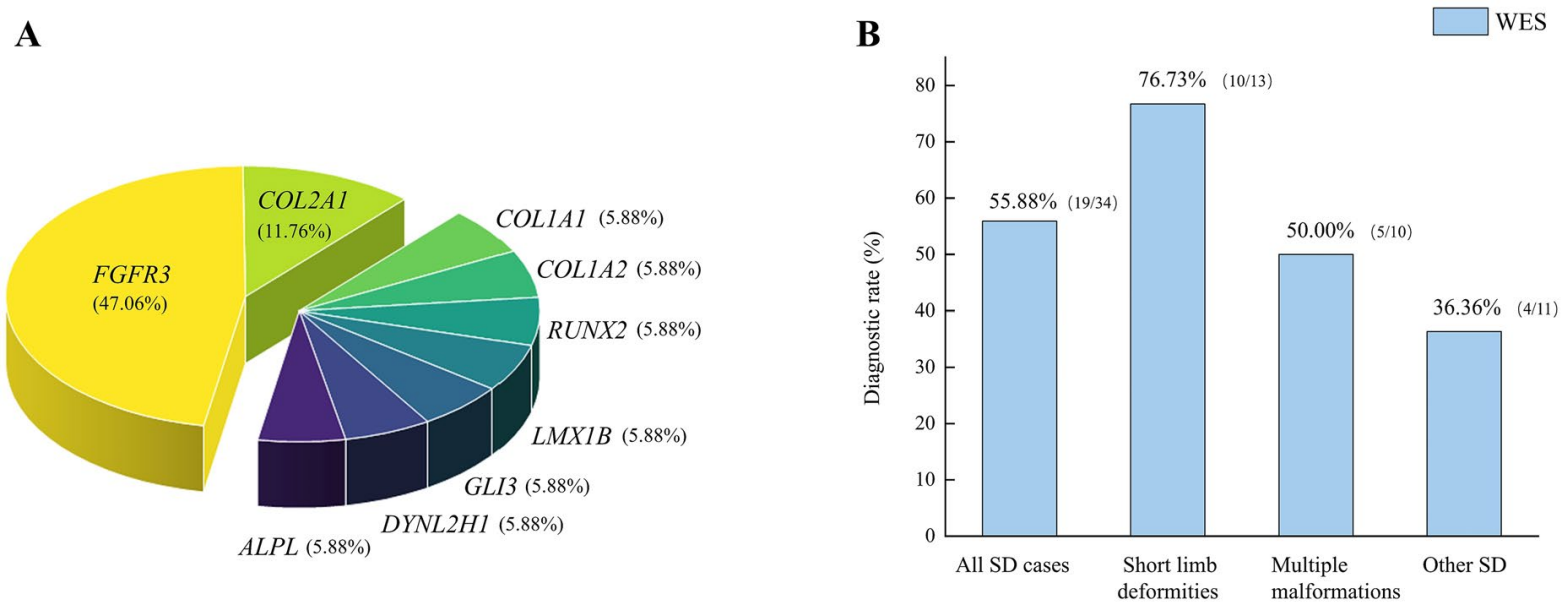
(**A**) Specturm of 9 genes in 17 positive fetuses using whole exome sequencing (WES). The most common gene was *FGFR3*, accounting for 47.06% (8/17), followed by *COL2A1* (22.76%, 2/17). (**B**) Diagnostic rate of WES in different groups divided by ultrasound features. All 34 SD cases were divided into three groups: 13 cases had short limb malformations (<-2SD, with or without abnormal shape of long bones, fracture or thoracic hypoplasia; 10 cases had multiple malformations; 11 cases were other SD such as polydactyly, syndactyly, talipes equinovarus, widened skull suture.

Of the 9 genes identified in the 17 positive fetuses with gene variations, 7 were AD inheritance pattern (*FGFR3*, *COL1A1*, *COL1A2*, *COL2A1*, *RUNX2*, *LMX1B*, *GLI3*), and 2 were AR inheritance pattern (*DYNC2H1*, *ALPL*). The *FGFR3* gene was the most prevalent SD-causing gene (47.06%, 8/17), followed by *COL2A1* (11.76%, 2/17) (Figure 2A). Among the 19 variations of the 9 genes, 11 were classified as “pathogenic” and 8 were classified as “likely pathogenic”. No novel variations were detected in our cohort.

Among three groups, the diagnostic rate of WES in short limb malformation group was the highest (76.3%, 10/13), followed by multiple malformations group (50%, 5/10), and other SD group (36.36%, 4/11) (Figure 2B). The diagnostic rate of WES in unilateral malformation group was 0% (0/4), while that in bilateral malformation group was 62.07% (18/29).

In 34 cases, total 27 cases (79.41%, 27/34) were performed not only WES, but also karyotyping and CMA. 18 cases (66.67%, 18/27) were found the genetic etiologies using those detecting methods. The detection rate of CMA and WES were 3.70% (1/27) and 62.96% (17/27), thus, WES increased the detection yield by 59.26%.

### 3.4. Validate the NIPD for the detection of skeletal dysplasia

Besides undergoing prenatal diagnosis through amniocentesis, 5 cases (Case 4, 6, 9, 11, and 12) also accepted the non-invasive prenatal diagnosis (NIPD) based on next-generation sequencing for the detection of skeletal dysplasia. Case 4 and Case 6 presented short and curved limbs, and the NIPD results showed that they all carried the *de novo* variation c.742C > T of *FGFR3* gene. The US abnormality of Case 9 was achondroplasia, short and curved limbs with “telephone receiver-shaped” changes, a distended abdomen, and an intracranial structural abnormality. The sequencing results showed Case 9 carried the heterozygous variation c.1948A > G of *FGFR3* which was inherited from the mother. Case 11 diagnosed with osteogenesis hypoplasia carried the heterozygous variation c.892G > A of *COL1A2* which was inherited from the mother. Case 12 presented short limbs and thoracic morphism was identified with the *de novo* variation c.2546G > T of *COL2A1*. These results were consistent to those obtained by sequencing amniotic fluid samples. The genotypes of five cases were correctly identified using NIPD based on NGS with no false-positive and false-negative results. The sensitivity and specificity of detecting SD by NIPD were 100 and 100%, respectively, in our study.

## 4. Discussion

In the present study, we sought to clarify the genetic etiologies of fetal skeletal dysplasia and evaluate the effects of CMA and WES. Using karyotyping and CMA, we successfully identified genetic etiologies in 3.70% SD fetuses. Using WES, we identified genetic etiologies in 55.88% cases. In addition, we carried out the non-invasive prenatal diagnosis (NIPD) based on next-generation sequencing (NGS) for the detection of skeletal dysplasia in 5 cases. Based on the genetic sequencing results, we analyzed the etiologies of SD, evaluated the risk of SD in the next pregnancy, and gave reasonable reproductive advice. Our study highlights the advantages of WES compared with CMA in genetic diagnosis in SD and provides the guidance for the reproductive advice. Furthermore, our study revealed the excellent detection efficiency of NIPD based on NGS, which may be a potential noninvasive detection method in fetal SD.

In our study, molecular testing of 34 fetal SD cases yielded a diagnostic rate of 55.88% (19/34). Our diagnostic rate is higher than 40.4% (38/94), 44% (12/27), 48% (31/64), and 50% (12/24) reported in previous reports on fetal SD in recent studies [14–17], but lower than 75% and 81.82% (45/55) [18,19]. This difference of diagnostic rate may be caused by inclusion criteria bias of the study, types of detection methods, and different sample sizes. Our 19 positive cases involved 2 cases with chromosomal abnormalities and 17 cases with variants of SD-associated genes. The diagnostic rate of chromosomal abnormalities in multiple malformations group (10%, 1/10) was higher than short limb malformations group (7.69%, 1/13), which is consistent with the previous reported studies [1,18]. It was reported that fetuses with multiple anomalies had a higher diagnostic rate using WES [20]. However, in our study, the diagnostic rate of WES in the short limb malformations group (76.3%, 10/13) is higher than the multiple malformations group (50%, 5/10). In addition, we observed a notably difference of diagnostic rate between the unilateral malformation group and bilateral malformation group (0 vs 62.07%). Nonetheless, prospective multicenter studies with larger sample sizes are needed to investigate the differences in detection rate of CMA or WES between the different clinical groups.

Fetal SD has high genetic heterogeneity and phenotypic diversity, which brings great challenges in prenatal counseling and pregnancy management for obstetricians and genetic counselors. Therefore, it is crucial to find a quick and accurate detection method for the diagnosis of fetal SD. So far, US is the most common test to detect fetal SD in the early pregnancy, and the diagnosis accuracy of continuous sequential ultrasound was over 80% [21,22]. Due to the overlapping features and phenotypic variability of SD, the ultrasound-only dependent test is inadequate for the diagnosis of fetal SD and impossible to differentiate the types of this disorder. Over the past decade, molecular detection technology like CMA or NGS has enhanced the identification of genetic causes of fetal SD [1]. Chromosomal aneuploid or microdeletion and microduplication were relatively rare, and the diagnostic rate of CNV-seq in fetal SD was low [17]. In our study, the diagnostic rate of CMA was 3.70%. Previous studies reported a significantly higher diagnosic rate of WES which was 70 ∼ 85% [23–25]. Recently, some studies have used WES to find the etiologies in fetal SD, which increased additional diagnostic yield to 32.7, 50, 75 and 77.8%, respectively [17,19,26,27]. In our cohort, WES increased the diagnostic yield was 59.26 compared with CMA. Our results revealed the high cost-effectiveness of WES in fetal SD. In the common molecular diagnostic process, the karyotyping analysis and CMA are usually the first-tier approach to detect chromosomal aneuploid and CNVs for fetal SD, which takes about 2 ∼ 3 weeks. If the results of CMA was normal, WES would be the next test step, which takes about 4 weeks again. Considering the time pressure and the diagnosis rate, we advised the pregnant women in the middle and third trimesters to accept CMA and WES for fetal SD at the same time. Thus, the whole process could be shortened to about 4 weeks, which provided parents more time in early parental decision making. For fetal abortion tissues, because parents usually did not need to make a decision as soon as possible, the cost-effectiveness of detection method was more considered rather than the detection time. Therefore, we thought that WES could be performed firstly to research the genetic cause of fetal abortion tissues. If the results of WES was normal, CMA would be carried out in the next step. The specific testing program should be designed according to the specific conditions of pregnant women to minimize the cost and maximize the benefit.

In the present study, a total 19 pathogenic or likely pathogenic variants affecting 9 genes, including *FGFR3*, *COL1A1*, *COL1A2*, *COL2A1*, *RUNX2*, *LMX1B*, *GLI3*, *DYNC2H1*, and *ALPL* were found to be associated with fetal SD. In a study by Huang et al. the most common genes were *FGFR3*, *COL1A1*, and *COL1A2*, while in another study reported by Zhang et al. the most common gene were *COL1A1*, *FGFR3*, and *COL2A1* [27,28]. In our study, the most common genes associated with the fetal SD was *FGFR3*, which accounted for 47.06%, followed by followed by *COL2A1* (11.76%) and *COL1A1* (5.88%). Our findings highlights the pathogenic or likely pathogenic variants in *FGFR3* and collagen genes are the most frequent genetic etiologies for SD, consistent with the results of other previous studies [29,30]. A Total 17 cases (89.47%, 17/19) were diagnosed as genetic SD including 39 different diaseaes, which classified into 16 groups according to the 2023 revision of the Nosology and Classification of Genetic Skeletal Disorders. The most common nosology groups in this study were the *FGFR3* chondrodysplasia group, the achondrogenesis and osteogenesis imperfecta and decreased bone density group, which was consistent with those previously reported [14].

Except for those 17 cases with variants of the above genes, the remaining two cases was identified with pathogenic CNVs. Case 1 with dup (11) (p15.5-p15.4) were diagnosed with Beckwith-Wiedemann syndrome (BWS), which was categorized into the group of 31 based on the Nosology and Classification of Genetic Skeletal Disorders. BWS is a genomic imprinting disorder characterized by overgrowth, macroglossia, abdominal wall defects, hemihyperplasia, and enlarged abdominal organs [31]. Approximately 43–65% patients showed overgrowth, and their both body weight and length were significantly higher than normal [32]. However, Case 1 had the opposite symptom (short limbs) without typical manifestations of BWS. Therefore, we considered that the CNVs dup (11) (p15.5-p15.4) might not be the genetic cause of short limbs malformations in Case 1, and the risk of recurrence in the next pregnancy is very low. In the end, the parents of Case 1 chose to terminate the pregnancy after the adequate genetic counseling. The Case 30 appeared short limbs and arrhythmia, and the results of WES showed a 56.82 Mb heterozygous deletion in the chromosome Xp22.33p11.21 (pathogenic, de novo) and a 98.10 Mb chimerism deletion (chimeric ratio 44%) in the chromosome Xp11.21q28 (pathogenic, de novo). The region of del(X)p22.33p11.21 contains 321 genes including *SHOX*, *USP9X*, and *ARSE*, which had the single dose effect. The deletion of *SHOX*, *USP9X* and *ARSE* could result in the irregular shortening of the middle limbs, short stature and overall development delay [33–35]. Therefore, we consider that the del(X)p22.33p11.21 was the genetic cause of Case 30. The region of del(X)p11.21q28 contains 509 genes including *ATP7A*, *AVPR2*, and *BTK*, which had the single dose effect. The deletion of *ATP7A*, *AVPR2*, and *BTK* could all cause the short limbs. Whereas the del(X)p11.21q28 was chimerism and the chimeric ratio was 44%, it was unclear whether this CNVs is related to the short limb malformations. Those two CNVs were all *de novo*, which are unlikely to recur in the next pregnancy. However, given the gonadal mosaicism in one of parents is possible, parents were still advised to performed the invasive prenatal diagnosis in subsequent pregnancy. The parents of Case 30 chose to terminate this pregnancy ultimately.

Nearly half of the cases could not obtain the definite genetic etiologies of SD, including four fetuses were identified with VUS variants. In Case 19 and Case 29, the VUS variant was inherited from mother or father who had a normal phenotype without skeletal abnormalities. Although those two VUS variants could partially explain some phenotypes, the evidence remained insufficient. Thus, we thought those VUS variants may not be the genetic cause of fetal skeletal dysplasia (SD). In Case 27, WES was performed on fetal tissue obtained after pregnancy termination. While we recommended Sanger sequencing of parents to validate this VUS variant, the parents declined further testing due to their own normal phenotypes. Based on the current available data, we cannot definitively determine whether this variant is responsible for the fetal SD. In Case 28, the c.3447_3464del variant of *COL2A1* gene was de novo. This variant was absent from the gnomAD database (PM2_P). This in-frame deletion in a non-repetitive region resulted in altered polypeptide length (PM4). According to ACMG guidelines, the variant was classified as VUS (PS2_M + PM2_P + PM4). In future studies, we will perform *in vivo* or *in vitro* functional assays or integrate multi-omics analyses to further evaluate the potential role of this variant, thereby helping clarify the genetic etiology of fetal SD. With an enrichment of genetic database and the accumulation of fetal phenotype-genotype database, the pathogenicity of those variants could be reassessed in the future and the genetic etiologies of those negative cases may be clear in the future. Some cases had no candidate variants detected in this study. It could be that the negative cases were caused by nongenetic factors such as environmental factors of drug exposure, or the causative variants may reside in the deep-intronic or non-coding regulatory regions, which could not be detected by WES. More studies such as whole genome sequencing (WGS) or genome methylation profile are warranted to explore the genetic etiologies of those fetuses.

Currently, the invasive testing is the gold standard for prenatal diagnosis. However, the risks of miscarriage and infection of the invasive detection deter many pregnant women. Here, we investigated whether NIPD based on NGS could be used for non-invasive prenatal detection and facilitate clinical diagnosis of fetal SD. A Total 5 cases accepted the NIPD based on NGS for the detection of fetal SD and the results were all consistent with those of prenatal diagnosis by amniocentesis. In previous studies, the noninvasive methods for fetal SD were limited to the *FGFR3* and *COL1A2* gene and only suitable for detecting *de novo* and paternal variants [36,37]. In this study, we detected the variants of three genes (*FGFR3*, *COL1A2*, and *COL2A1*) using NIPD based on NGS and also analyzed the status of maternally inherited variants *via* molecular counting techniques to determine the allele frequency of fetuses, which showed wider range of genes and variety of variants. In Case 11, the fetus diagnosed with osteogenesis hypoplasia carried the heterozygous variation c.892G > A of *COL1A2*, which inherited from mother. In the second pregnancy, we tried to detect this maternally inherited variant by NIPD based on NGS and the results showed the genotype of the fetus was wild type, which was also confirmed by the invasive prenatal diagnosis. Our results demonstrated that NIPD based on NGS is a promising tool for the noninvasive detection of fetal SD in early gestation weeks. Undoubtedly, more validation researches with larger samples are still required to determine the accuracy and the clinical utility of NIPD based on NGS for fetal SD.

There are still some limitations in our study. First, our sample size was relatively small, thus our findings based on this single-center study cannot be generalized to the broader pregnant women with fetal SD. Second, some cases was only performed WES after abortion without CMA due to the refusal of the parents. Third, besides pathogenic or likely pathogenic variants, we also identified some variants considered as VUS. Further assessment and functional study should be carried out to analyze the effects of those variants on fetuses.

## 5. Conclusions

In summary, the diagnostic rate in our study was 55.88%, and the high increased detection rate of WES compared with CMA indicated that WES is essential in fetal SD. In addition, of the 19 positive fetuses, 2 fetuses were identified with pathogenic CNVs, and other 17 fetuses were identified with pathogenic or likely pathogenic variations. Total 19 variations of the 9 genes were identified, of which zero were novel. Moreover, we carried out the NIPD based on NGS for the detection of fetal SD in 5 cases and results were consisent with amniocentesis, which showed this method was promising and applicable. Our findings not only enriched the phenotypic and genotypic data of fetal SD, but also provided appropriate genetic counselling and reasonable advise on decision making for affected families.

## Supplementary Material

Figure S2.JPG

Figure S1.JPG

Supplementary Table.xlsx

Figure S4.JPG

Figure S3.JPG

Supplementary Figure.docx

## Data Availability

The data that support the findings of this study are available from the corresponding author upon reasonable request.
