# Supplementary figures and images for: Genetic analysis of fetal skeletal dysplasia via whole exome sequencing and non-invasive prenatal diagnosis
Source: Ann Med. 2025 Dec 28;58(1):2606517. doi: 10.1080/07853890.2025.2606517 (PMC12777751; doi:10.1080/07853890.2025.2606517)

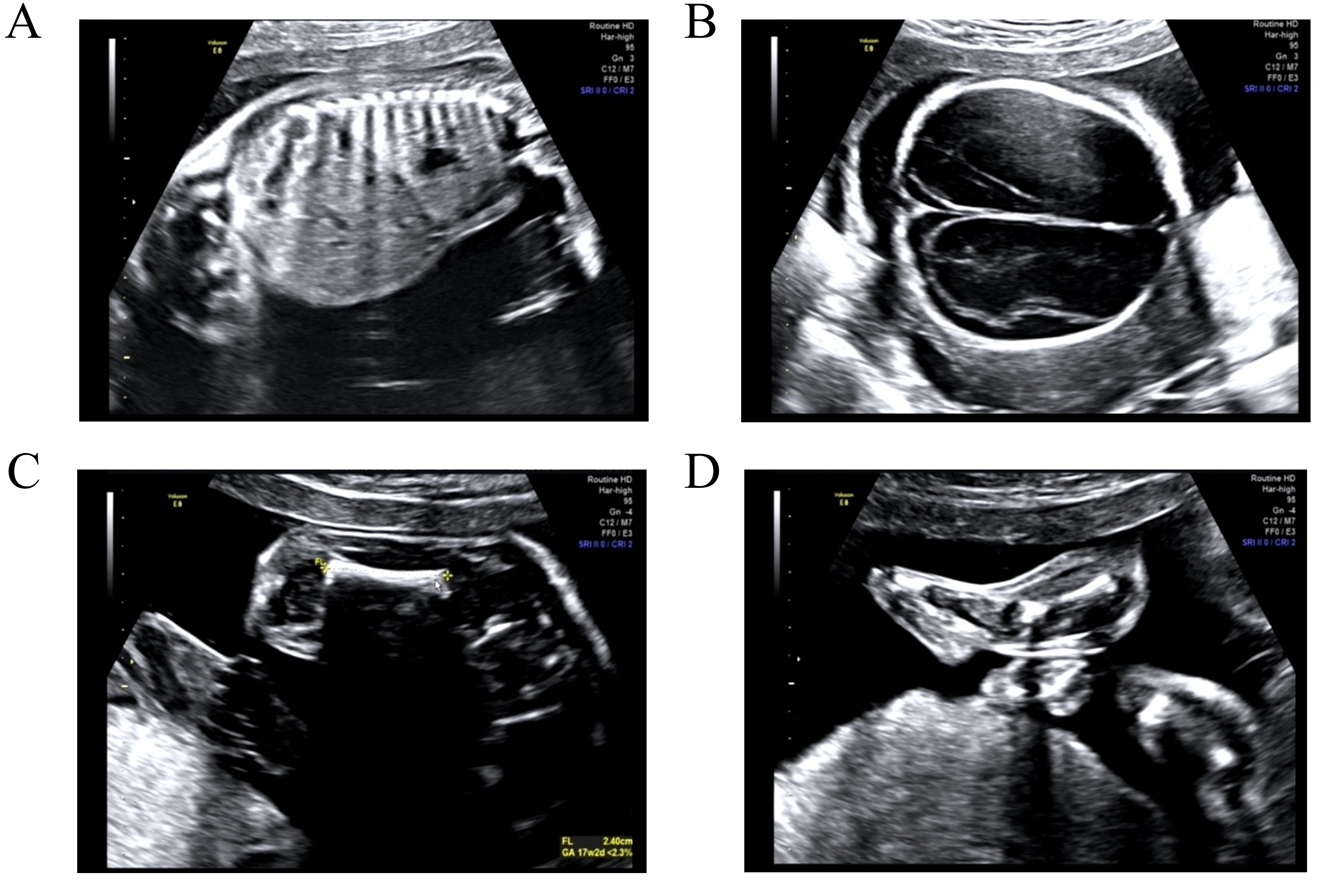

Supplement: Figure S2.JPG [file IANN_A_2606517_SM3963.jpg]

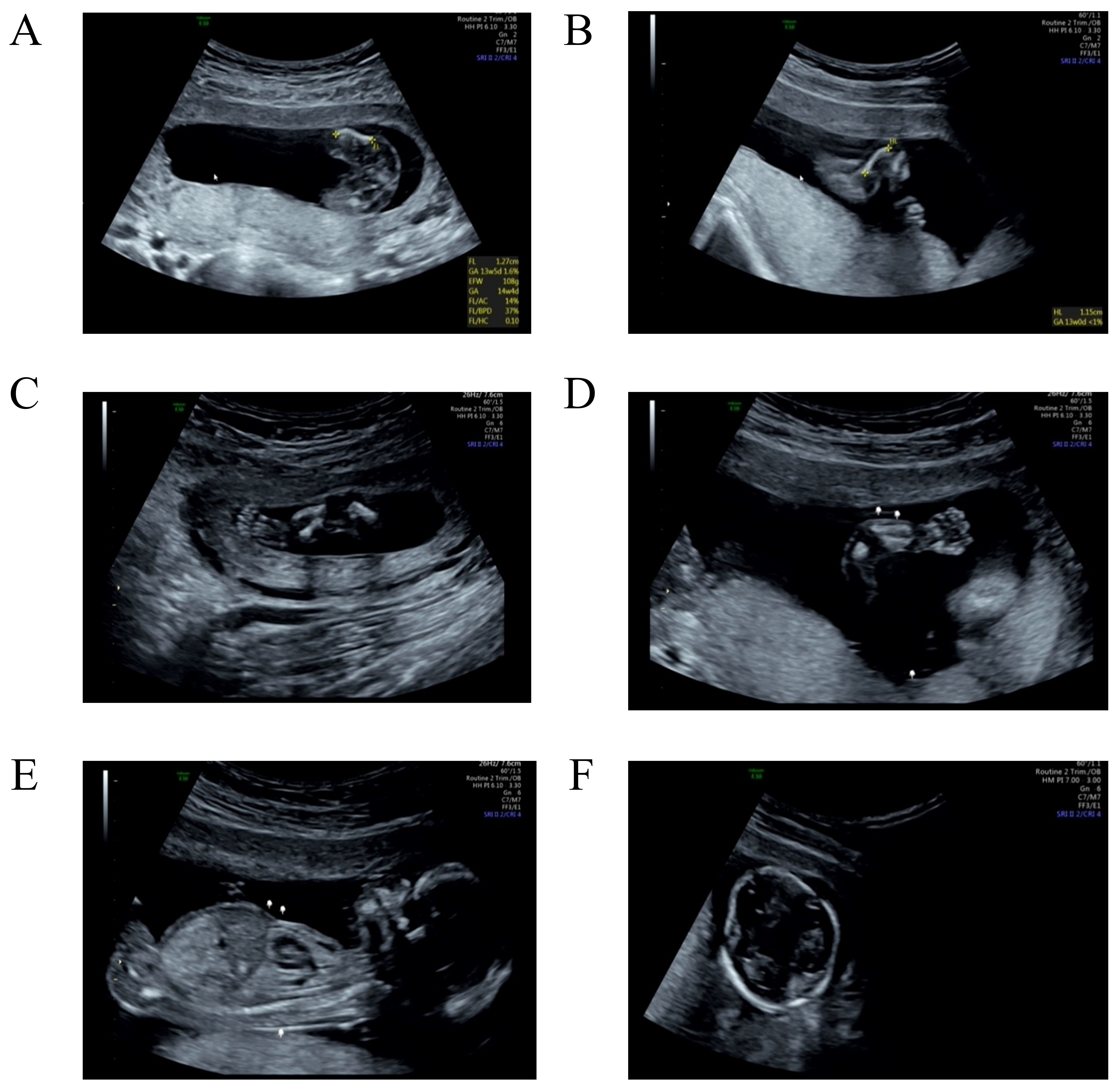

Supplement: Figure S1.JPG [file IANN_A_2606517_SM3962.jpg]

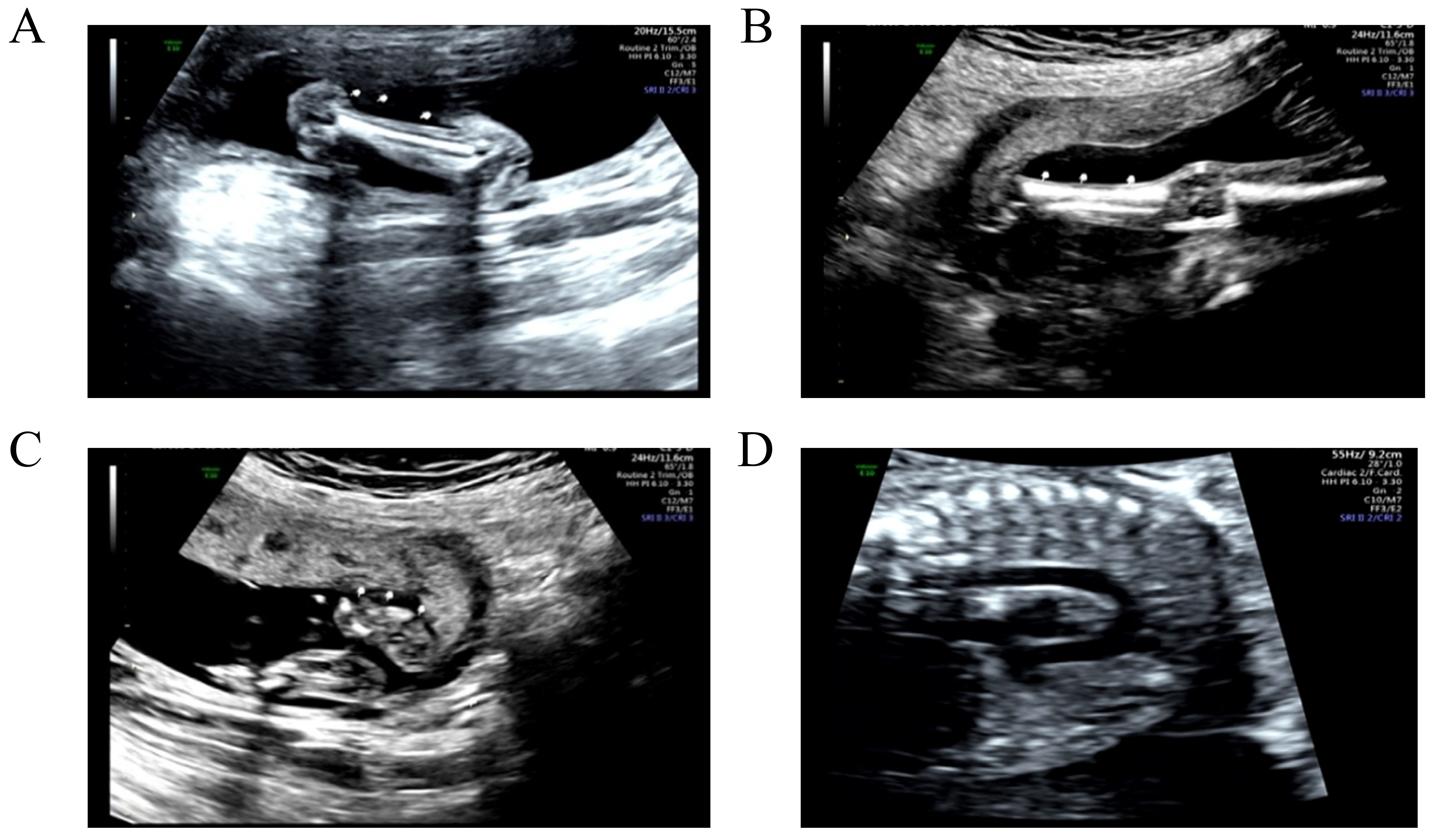

Supplement: Figure S4.JPG [file IANN_A_2606517_SM3960.jpg]

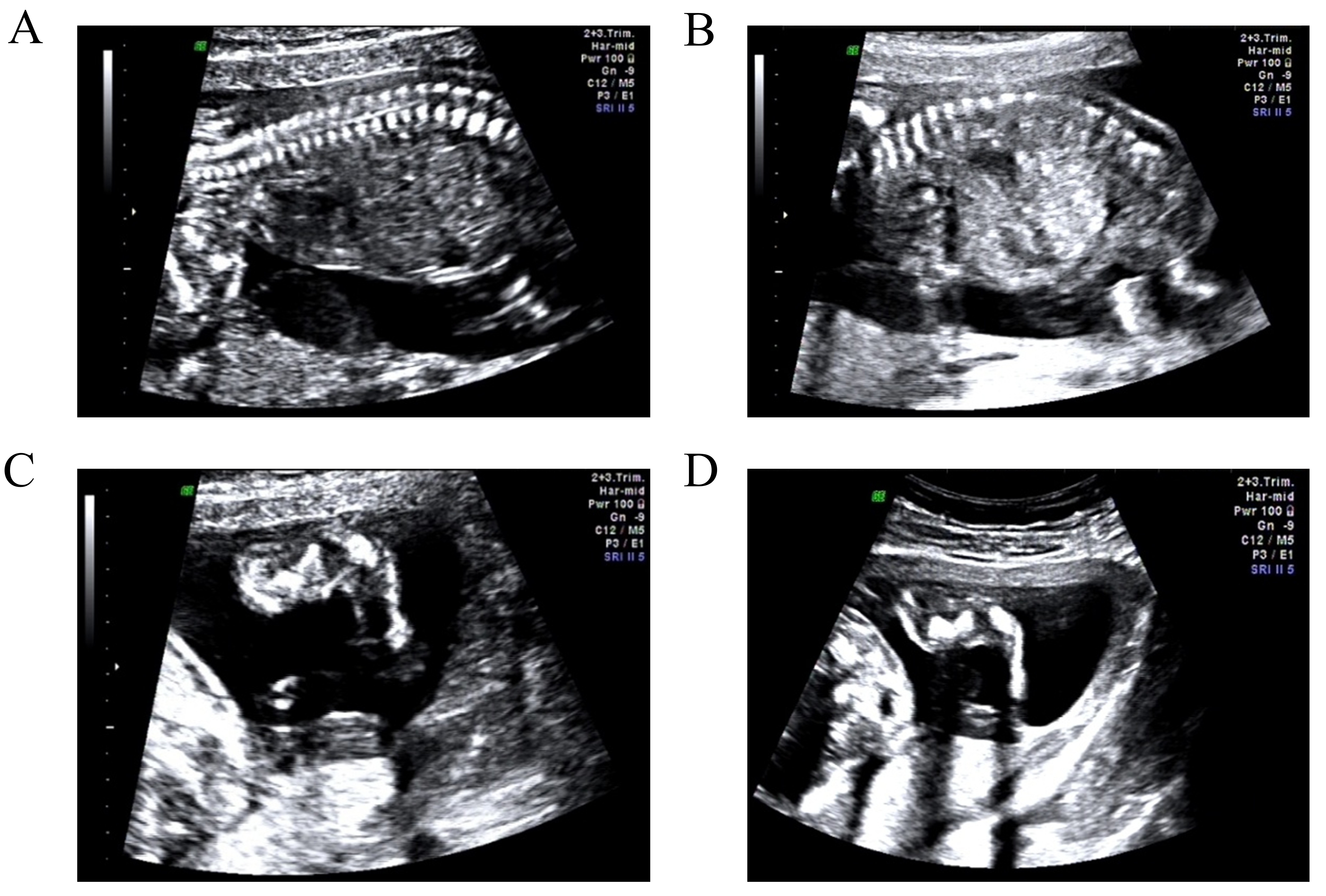

Supplement: Figure S3.JPG [file IANN_A_2606517_SM3959.jpg]
