# Supplementary material for: Genetic analysis of fetal skeletal dysplasia via whole exome sequencing and non-invasive prenatal diagnosis
Source: Ann Med. 2025 Dec 28;58(1):2606517. doi: 10.1080/07853890.2025.2606517 (PMC12777751; doi:10.1080/07853890.2025.2606517)
Supplement: Supplementary Figure.docx [file IANN_A_2606517_SM3958.docx]

Images of US examination of some fetuses with skeletal dysplasia in our study.


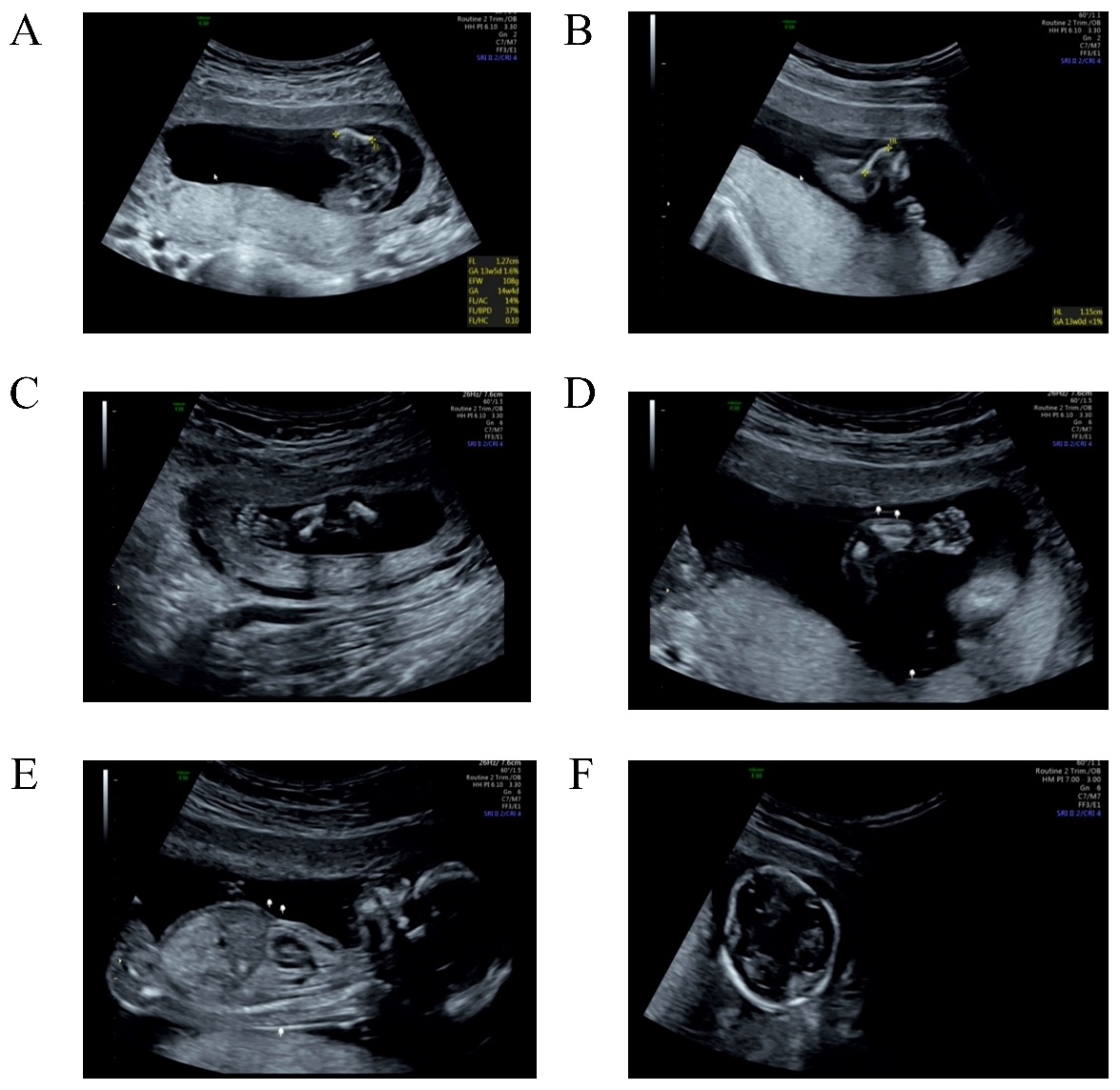


Figure S1. Images of ultrasound examination in case 5 (*FGFR3* c.742C>T) (A) short and curved femur; (B) short and curved humerus; (C) short tibia; (D) short radius; (E) narrow thorax; (F) strawberry-shaped skull ring.


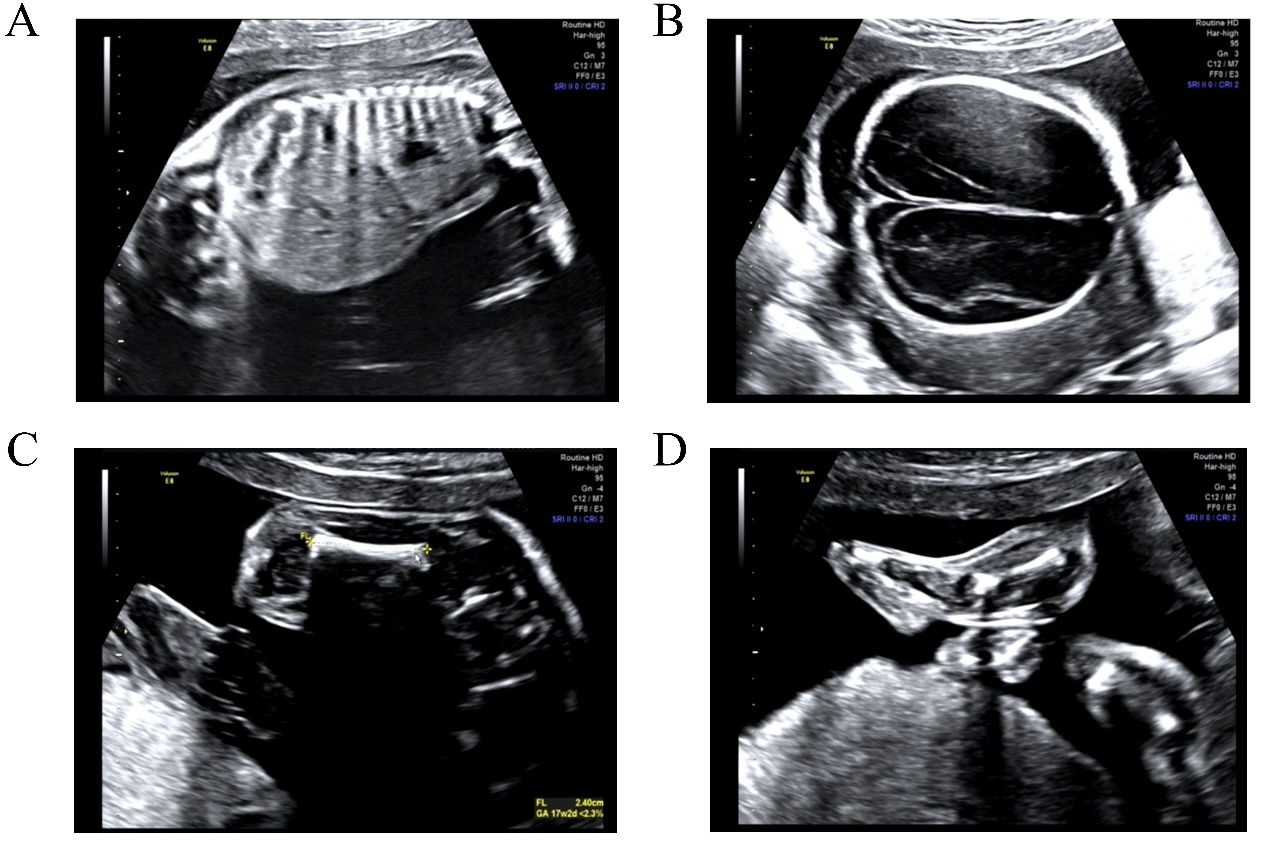


Figure S2: Images of ultrasound examination in case 9 (*FGFR3* c.1948A>G). (A) distended abdomen; (B) intracranial structural dysplasia; (C) and (D) achondroplasia (short and curved limbs).


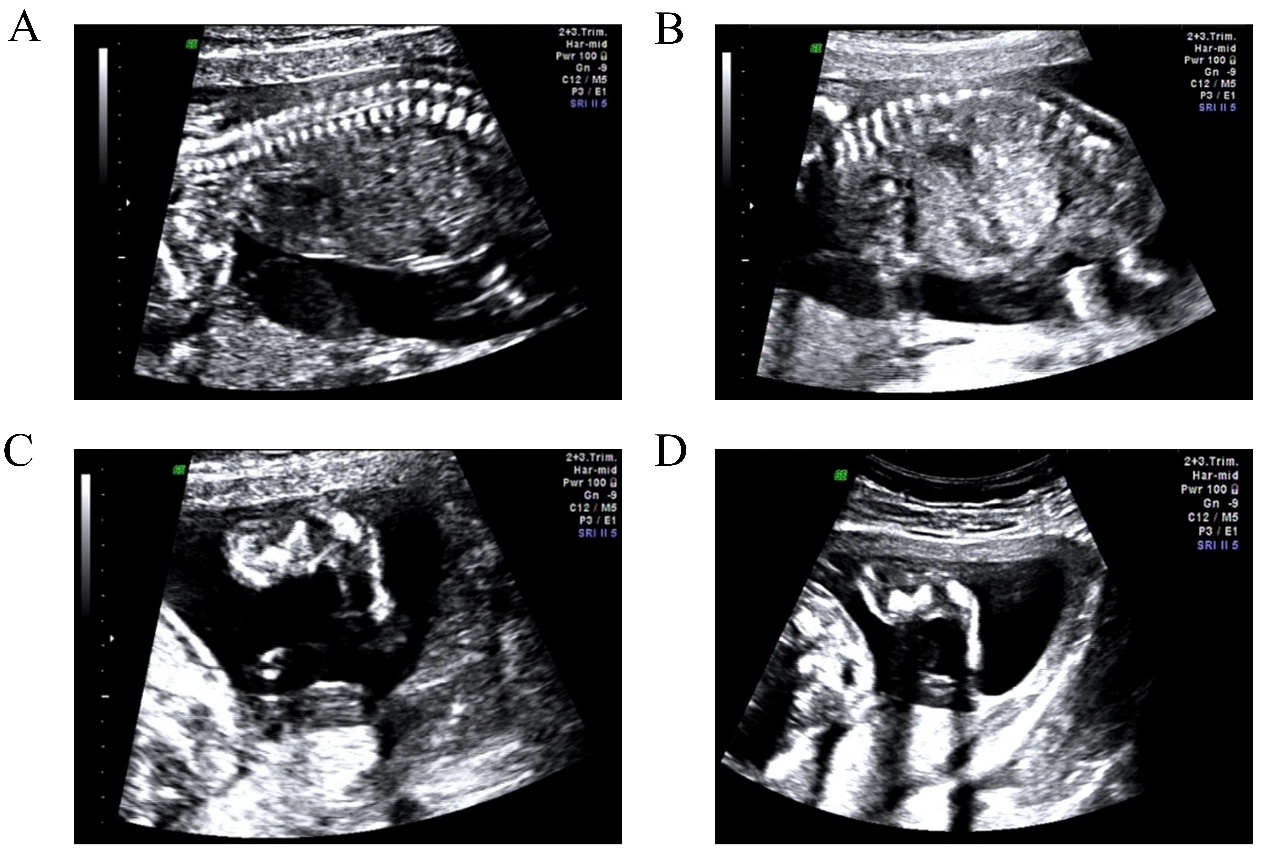


Figure S3: Images of ultrasound examination in case 10 (*COL1A1* c.2110G>C). (A) and (B) narrow thorax; (C) and (D) short femur.


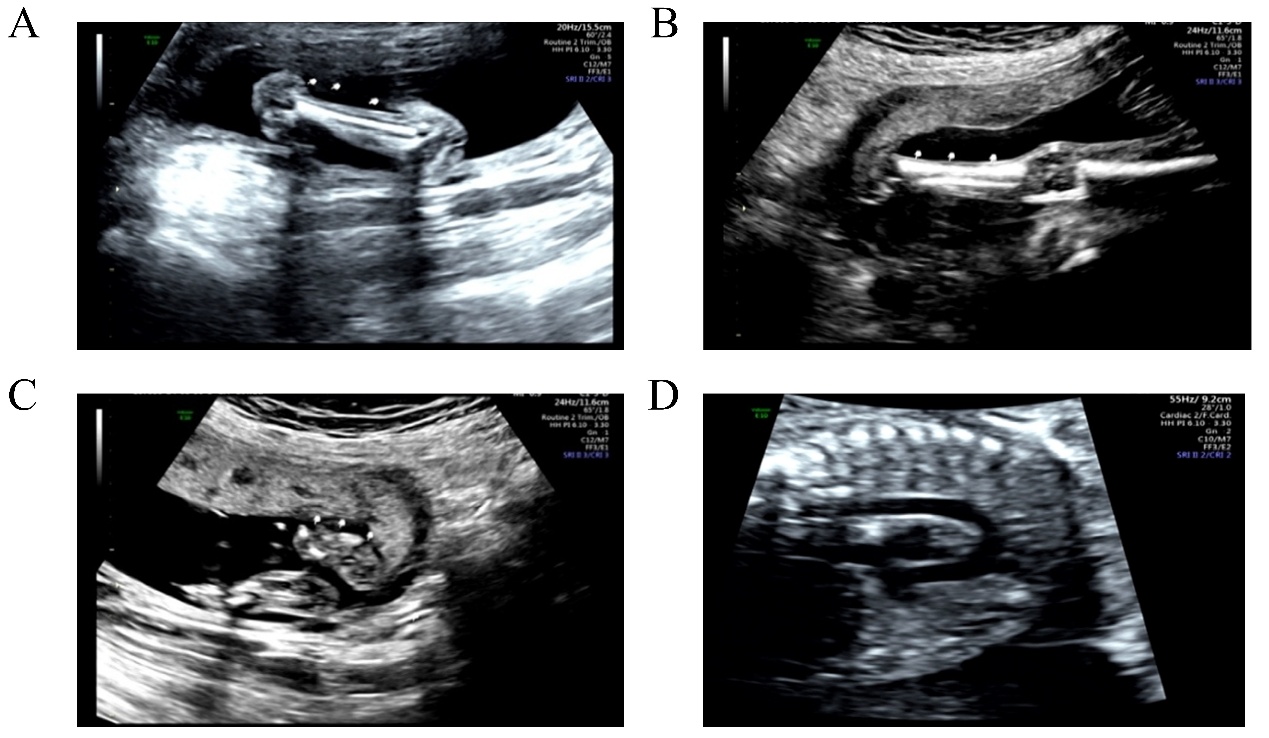


Figure S4: Images of ultrasound examination in case 34 (negative results of WES). (A) and (B) short left leg and reduction of subcutaneous tissue; (C) short left foot in the shape of a rocking chair; (D) fetal bovine aortic arch.
